# A Comparison between the Predictors of Vaccine Uptake Intentions for Influenza and Dengue

**DOI:** 10.3390/ijerph15122694

**Published:** 2018-11-29

**Authors:** Chitra Panchapakesan, Anita Sheldenkar, Ysa Marie Cayabyab, Janelle Shaina Ng, Jiahui Lu, May O. Lwin

**Affiliations:** Nanyang Technological University, Wee Kim Wee School of Communication and Information, Singapore 637718, Singapore; anitas@ntu.edu.sg (A.S.); ysacayabyab@ntu.edu.sg (Y.M.C.); janelleng@ntu.edu.sg (J.S.N.); jhlu@ntu.edu.sg (J.L.); tmaylwin@ntu.edu.sg (M.O.L.)

**Keywords:** vaccine uptake intentions, influenza, dengue

## Abstract

Infectious diseases have a huge health and economic burden globally. Vaccination has been found to be a crucial health intervention for diseases. The study aims to compare the drivers of vaccine uptake for influenza and dengue, and to understand the key drivers within each of the diseases in predicting vaccine uptake intentions. Data were collected from 1000 Singaporeans and Singapore permanent residents between the ages of 21 and 70 through face to face surveys. Overall, intention to get vaccinated was low for both diseases. Comparing the means between the knowledge and perceptions regarding influenza and dengue and their vaccine uptake intentions, perceived susceptibility was significantly higher for influenza than dengue; and perceived severity, knowledge of the disease, and benefits of the vaccine were significantly higher for dengue. Looking at the key drivers within the diseases, perceived susceptibility towards the diseases and benefits of the vaccine were positively associated with vaccination uptake intentions for influenza and dengue. Perceived barriers towards the vaccine were negatively associated with vaccine uptake for dengue. Programs conducted for public health promotion should focus on increasing the public’s awareness of the susceptibility and seriousness of the diseases, and the benefits of getting vaccinated.

## 1. Introduction

Globally, infectious disease outbreaks have increased steadily over the last 30 years, contributing significantly to the global burden of disease [1]. One such infectious disease is seasonal influenza. An estimate of 290,000–650,000 deaths occur annually around the world due to influenza-associated respiratory diseases [2]. Another perennial and severe disease in tropical countries is dengue fever. Globally, an estimated 3.9 billion people are at risk of contracting the disease [3], and Southeast Asia alone has an estimated economic burden of USD 950 million each year [4].

As a city state hosting high volumes of international travelers [5], Singapore’s population is continually at risk of exposure to imported infectious diseases. As such, it also has the added potential of becoming a hub for exporting infectious diseases to other places around the globe. Singapore, being close to the Equator, is also at risk of both the southern and northern strains of seasonal influenza, and susceptible to the virus all year round [6]. The country is also at risk of dengue. Over the last few decades, the frequency and magnitude of dengue fever has increased in tandem with the increased density of Singapore’s urban population [7]. In response, the Singapore government has implemented regular initiatives and campaigns for dengue prevention [8,9].

Immunisation through the administration of vaccines has long been acknowledged as a cost-effective and crucial health intervention among the general population by building herd immunity [10]. The World Health Organisation (WHO) [11] has acknowledged that vaccinations prevent approximately 2–3 million deaths per year from a host of potentially dangerous but preventable diseases. To encourage vaccinations, WHO has formulated a Global Action Plan (GAP) which calls for a global increase in seasonal vaccine use to mitigate the burden of seasonal influenza worldwide, as well as to increase global preparedness levels against pandemics [12,13].

Whilst vaccination is an effective preventive measure against influenza [14], developed countries such as the United States, the United Kingdom, and Japan have low vaccination uptake rates, ranging from approximately 30–40% [15,16]. In Singapore, influenza vaccinations of residents through general hospitals and private clinics are recommended, especially for high risk populations like the elderly and young children. Despite this ease of access, the uptake rate has been low at merely 15.2% in 2013 [17]. Various reasons have been proposed to explain these low uptake rates. For example, studies have suggested that individuals who perceive themselves as being less susceptible to influenza and who have misconceptions on the potential severity of the disease tend to remain unvaccinated [16,18]. In short, uptake of vaccinations across populations is generally low unless the perceived severity surrounding the disease is high [19].

For dengue, the vaccine Dengvaxia, which was first distributed in 2017, has also been approved and made accessible in Singapore [20]. However, due to factors relating to the recency of this vaccine, it has not been as well promoted as the influenza vaccination, and no data has yet been released on the uptake rate within Singapore. Singapore is more often placed on high alert with frequent news coverage on new dengue clusters [21] and deaths due to dengue fever [22]. This contrasts with the relatively quiet reporting on influenza. As such, the threat of dengue fever is often a more salient concern among the Singapore population.

If Singapore were able to achieve high rates of immunisation, the resulting herd immunity effect would make it more resilient against preventable pandemics. This would also deny infectious diseases the opportunity to use Singapore as a staging ground to infect other parts of the world. In light of the potential to prevent and deny infectious disease access through Singapore via high vaccination rates, it is imperative that we understand the drivers of vaccine uptake which can improve vaccination rates to a suitable level.

Hence, this research proposes to understand the factors that predict vaccine uptake intentions for two major diseases—influenza and dengue. The following research objectives are addressed:

*Research objective 1*: To compare the knowledge and perceptions of influenza and dengue and their respective vaccines.
*Research objective 2*: To understand the key drivers within each of the diseases in predicting vaccine uptake intentions.

This study attempts to make a within-subjects comparison of the drivers of vaccine uptake for influenza and dengue fever among Singaporeans and Singapore permanent residents. Both diseases are well-known and recur frequently and have been the subject of targeted health campaigns (Influenza [23]; Dengue [8]). Therefore, both diseases are comparably salient and the perceived knowledge about them is relatively high. However, because dengue fever causes deaths more directly in vulnerable populations than influenza, dengue fever is expected to be perceived as a more serious illness. This study further compares the Health Belief Model (HBM) constructs of susceptibility, perceived barriers, and perceived benefits as predictors of intention to vaccinate. To our knowledge, no study has attempted a within-subjects comparison of disease perception in predicting vaccination uptake.

## 2. Methods

### 2.1. Study Design and Sample

Face to face survey interviews were conducted amongst Singaporeans and Singapore permanent residents between the ages of 21 and 70. Address listings were first obtained from the Department of Statistics (DOS) to give a fully randomized and representative sample of households from different housing types and geographical locations within Singapore based on the Urban Redevelopment Authority’s (URA) planning areas [24]. The DOS tracks the addresses that have been used by other studies and prevents them from being re-used for a certain period of time, thus minimising the risk of survey fatigue. The obtained address listings were then sampled from a broad, representative distribution of dwelling types within those geographical regions.

One thousand respondents were recruited. The data was manually combed to identify any ambiguous or erroneous responses. If issues or mistakes were found within the data, respondents were telephoned to clarify responses. Thirty percent of all respondents were also randomly telephoned to verify the data. Individuals who did not meet the criteria (i.e., not between the ages of 21 and 70 or not a Singaporean national or permanent resident) or could not be included due to missing data (*n* = 48), were excluded from the analyses. New respondents were obtained to meet the target sample of 1000. The response rate for the first data collection period was 43.4%, and 11.1% for the second set. All subjects gave their informed consent for inclusion before they participated in the study. The study protocol was approved by the Institutional Review Board of Nanyang Technological University, (IRB-2016-05-016).

### 2.2. Data Collection

Interviewers were trained to ensure that they were familiar with the questions and that the questions were asked in a standardised format. Interviewers read out the questions and provided choice answers to participants, who then selected their answers to be recorded by the interviewer. Responses were entered into a Computer Assisted Personal Interviewing (CAPI) digital survey platform designed with logic features to reduce the likelihood of errors and to ensure all questions were answered. Incentives were given in the form of 11 USD shopping vouchers. Interviewers were also randomly shadowed by a study supervisor to ensure protocol was adequately executed. If the original target household was not available, interviewers were instructed to move to the housing unit one floor below or, if within a landed property, move to the next house along the street to make sure the dataset was representative of the population.

### 2.3. Survey Design and Measures

The survey was designed in English—the predominant language spoken within Singapore— and then translated into Mandarin and Malay, which are the two other major languages within the country. The survey was then back-translated into English to check for accuracy. The questionnaire was piloted by both the survey designers and the interviewers to ensure internal validity, assess question wording, and to evaluate the interviewer.

The questions focused on perceived susceptibility and severity to influenza/dengue, barriers and benefits to influenza/dengue vaccination, perceived knowledge of the diseases; and demographics. The measures were created based on the Health Belief Model, which posits that perceived susceptibility, severity, and benefits and barriers to action, have been found to be important predictors of health behaviour; in this case—vaccination [25]. This is supported by Brewer and colleagues’ meta-analysis [26], which found that perceived susceptibility and severity showed strong positive association with the likelihood to vaccinate. Each measure was constructed by averaging the score of the composite questions and then tested for reliability using Cronbach’s alpha.

Vaccine uptake intention was measured with the question, “How likely are you to get vaccinated against influenza in the next year?” using a five-point Likert scale ranging from 1 (Definitely won’t) to 5 (Definitely will). Perceived severity of the diseases were similarly measured on a five-point Likert scale ranging from 1 (Strongly disagree) to 5 (Strongly agree) using the following items modified from Kwong et al. [27]: (a) “Influenza/dengue is a serious illness.”, (b) “Complications from influenza/dengue are serious.” and (c) “I will be very sick if I get influenza/dengue.”. Internal consistency of the scales were high for both influenza (*α* = 0.89) and dengue (*α* = 0.90).

Perceived susceptibility of influenza (*α* = 0.92) and dengue (*α* = 0.93) were measured with three items modified from Kwong et al. [27]: (a) “I think I am at high risk of getting influenza/dengue.”, (b) “My chances of getting influenza/dengue in the next few months are great.” and (c) “I am more likely than other people to get the flu/dengue.”.

Perceived benefits of vaccines were measured with three items for influenza (*α* = 0.87) and dengue (*α* = 0.90), modified from Cheney et al. [28]: (a) “I would rather have a flu/dengue vaccine than get the flu/dengue.”, (b) “If my doctor/nurse recommends taking an influenza/dengue vaccination, I will take it.” and (c) “I would get the influenza/dengue vaccine to stay healthy.”.

Perceived barriers of vaccines were measured with three items for both influenza (*α* = 0.84) and dengue (*α* = 0.68), modified from Cheney et al. [28]: (a) “I worry about side effects from the influenza/dengue vaccine.” (b) “Healthy people do not need to get an influenza/dengue vaccine.” and (c) “I wonder about the safety of the influenza/dengue vaccine.”.

Perceived knowledge has also been found to significantly impact health behaviour in numerous studies [29] and has therefore been included within this study. Knowledge was measured using three items: (a) “I am very knowledgeable about Influenza/dengue.” (b) “Compared to most people, I am well informed about the ways to prevent influenza/dengue.” and (c) “I know a lot about the symptoms of influenza/dengue.” for both influenza (*α* = 0.89) and dengue (*α* = 0.93). The questions were constructed using validated scales from Centers for Disease Control and Prevention (CDC) [30] and National Environment Agency (NEA) [31].

### 2.4. Statistical Analysis

Paired samples *t*-tests were used to compare health belief variables between the diseases. Multivariate analyses were used to investigate associations between predictors (perceived severity, perceived susceptibility, barriers and benefits to vaccination) and vaccine uptake, and control for demographic variables (age, gender, ethnicity, educational attainment). For all analyses, a *p*-value of ≤0.01 was considered significant because of multiple comparisons [32] and the relatively large sample size. Statistical analysis was performed using STATA 11.2 (StataCorp, College Station, TX, USA) and SPSS version 12.0 (IBM, Armonk, NY, US).

## 3. Results

Among the respondents recruited, the mean age was 43.41 (SD = 14.65), and 61.3% were females. Table 1 presents the demographic characteristics of the participants. Reflecting the Singapore population, the majority were of Chinese ethnicity (78.5%) and have at least completed General Certificate of Education (GCE), O (Ordinary)/N (Normal)/A (Advanced) level or Diploma (56.6%) (See Table 1).

Only 23.7% of respondents reported that they would probably or definitely intend to vaccinate in the next year against influenza, while 21.0% said they would probably or definitely vaccinate against dengue. The majority of respondents were undecided as to whether they intend to get vaccinated against influenza and dengue (41% and 49.5% respectively).

Perceived susceptibility was low for both diseases. A mean score of 2.32 (SD = 0.90) and 2.22 (SD = 0.89) was found for influenza and dengue, respectively, with respondents perceiving themselves to be significantly more susceptible to influenza than dengue. The mean for perceived severity and benefits to getting vaccinated, on the other hand, were higher for both diseases. The mean scores for influenza and dengue were 3.18 (SD = 0.98) and 3.99 (SD = 0.87) for perceived severity, and 3.54 (SD = 0.94) and 3.69 (SD = 0.91) for perceived benefits, respectively. For both predictors, however, dengue was significantly scored higher than influenza. The perceived barriers to both influenza and dengue vaccine uptake were 3.07 (SD = 0.88) and 3.00 (SD = 0.86), respectively, and they were not significantly different. Lastly, in terms of knowledge of the disease, the participants perceived themselves as knowing significantly more about dengue (M = 3.17, SD = 0.84) than influenza (M = 2.87, SD = 0.87), although both scores were somewhat average (See Table 2).

To understand how these factors affected the two diseases separately, multiple regression analyses were conducted to determine the relationship between the five predictors (severity, susceptibility, benefits, barriers, and knowledge) of vaccine uptake and the intention to get vaccinated in the following year for influenza and dengue. To fully assess the extent of influence of the independent variables, gender, age, ethnicity, and educational attainment were controlled for. Results show that the five predictors explained a significant proportion of the variance (R^2^ =16.7%). Specifically, intention to get vaccinated for influenza was significantly predicted by higher perceived susceptibility (*β* = 0.25, *t* = 7.93, *p* < 0.001), and higher perceived benefits of getting vaccinated (*β* = 0.31, *t* = 8.95, *p* ≤ 0.001). However, perceived susceptibility of the disease, knowledge of the disease, and perceived barriers of getting vaccinated were not significant predictors of vaccine uptake (See Table 3).

On the other hand, for dengue vaccine uptake, regression analyses show that the model explain 18.9% of the total variance. Specifically, higher perceived susceptibility (*β* = 0.28, *t* = 9.02, *p* ≤ 0.001), higher perceived benefits of getting vaccinated (*β* = 0.32, *t* = 9.29, *p* ≤ 0.001), and lower barriers to getting vaccinated (*β* = −0.13, *t* = −4.13, p ≤ 0.001) significantly predicted behavioural intention to vaccinate. Severity and knowledge of dengue were not found to significantly predict intention to vaccinate (See Table 4).

## 4. Discussion

This is one of the first studies conducted in Singapore that examines factors influencing the potential uptake of vaccines for two different diseases—influenza and dengue—using a within-subject design. Influenza and dengue are two major diseases that affect the Singapore population. Comparing factors such as susceptibility, severity, and knowledge of the diseases and benefits and barriers of the vaccines can help in understanding the way the public perceive these diseases and their vaccination uptake intentions. Overall, intention to get vaccinated was low for both diseases, with only approximately one fifth of the participants indicating that they would consider getting vaccinated against influenza and dengue the following year.

The study shows that perceived susceptibility to influenza and dengue were low, suggesting that participants do not consider themselves to be at high risk of getting the diseases. However, perceived susceptibility was significantly higher for influenza than dengue, indicating that participants think they are more likely to get influenza than dengue. One major reason could be because influenza is more prevalent in Singapore. Official sources stated that in 2017, thirteen people per day sought treatment for influenza from polyclinics [33], while only ninety-one people have been diagnosed with dengue within the year [34]. Also, this study found that approximately three times as many people had been diagnosed with influenza in the last year, in comparison to those who had ever been diagnosed with dengue. More people are aware of others who have been diagnosed with influenza and may therefore consider themselves more susceptible to it. In addition, since dengue is a vector borne disease, individuals could be taking active measures such as applying mosquito repellent sprays or avoiding high mosquito breeding areas in order to ensure their safety. At the same time, individuals may feel uncertain of how to protect themselves from influenza as they cannot predict how and when they will get it during social interactions.

Perceived severity had the highest score of all the predictors for dengue suggesting that dengue is seen to be a dangerous disease. Severity was also significantly higher for dengue than influenza, which was predominantly seen as only moderately severe. This could be because Singapore is more often placed on high alert about deaths due to dengue fever [22]. This contrasts with the frequent news coverage depicting influenza as a mild disease. Perceived benefits of getting vaccinated were moderately high for both influenza and dengue, implying that getting vaccinated is perceived as effective in reducing the risks of getting the disease. In addition, getting vaccinated against dengue is seen as significantly more beneficial than getting vaccinated against influenza, potentially due to the higher perceived severity of dengue, which may suggest that more action is needed to prevent the disease.

Both barriers to getting vaccinated and perceived knowledge of the diseases had middling mean scores, indicating that the participants were neutral towards the tangible and psychological costs of getting vaccinated, and their perceived knowledge of the diseases were not better or worse than others. However, there were no significant differences in barriers towards getting vaccinated against influenza nor dengue.

The discussion so far indicates that there are significant differences between influenza and dengue for the five factors generally associated with vaccine uptake, thus answering the first research objective of this study. To further understand the effect of the factors that are associated with the intention to vaccinate for both the diseases, multiple regression analysis was attempted for each disease.

In the case of influenza, higher perceived susceptibility and perceived benefits of the influenza vaccine were positively associated with a higher intention of getting vaccinated. Benefits of getting vaccinated and susceptibility to influenza seem to be strong predictors of individuals’ intention to vaccinate. This is in line with other studies that have reported vaccinated participants feeling more susceptible to the disease, and perceiving more benefits from the vaccine and less barriers to take the vaccine [35].

Perceived severity, knowledge of the disease, and barriers to vaccination did not have any significant relationship with the likelihood of getting the influenza vaccine. This contradicts previous studies that have found severity and knowledge to be predictors of vaccine uptake [19,27]. However, studies also suggest that the predictive power of these drivers were relatively low [26,36,37]. Some studies have also found that barriers may not be a direct predictor of vaccine uptake but may be a moderator [38]. Future studies should investigate these relationships further.

In the case of dengue, the results mirror those found in influenza, with perceived susceptibility of the disease and benefits of the vaccine being positively associated with the likelihood of getting vaccinated against dengue. In addition, the higher the perceived barriers to vaccination, the less likely respondents are to get vaccinated. This is in line with other studies [39]. Although a vaccine for dengue (Dengvaxia) has been available since 2017 and has been approved for use within Singapore, the vaccine is only significantly effective against two of the four strains of dengue that are not primarily found in Singapore; and the risk of contracting a more severe form of the disease is reportedly higher if there is no prior exposure to the disease before vaccination [9]. Therefore, promotion of the vaccine has been minimal and public awareness is thought to be low, which could be a reason why barriers were significantly negatively related to uptake. Increasing public education on these preventative measures are needed in the future.

Severity and knowledge of dengue did not have any significant relationship with the likelihood of getting dengue vaccine, which is similar to the results found while investigating influenza. This could be the result of the contrast between severity being perceived as high for dengue, but susceptibility being perceived as fairly low. As preventative measures such as the “Mozzie wipeout” [40] are promoted heavily within Singapore, people may think they are not at risk of getting dengue and thus opt to adopt preventative measures against mosquito breeding rather than vaccination methods. This answers this study’s second research objective. To summarise, the discussion shows that despite the differences in perceptions between the diseases, factors such as susceptibility, severity, and knowledge of the diseases and benefits and barriers of the vaccines do play a vital role in predicting the public’s intention towards vaccine uptake for both diseases.

Finally, there are few limitations to the study. While cues to action is a concept included in the HBM model, it was not considered because the main objective of this study was to assess personal factors that drive behavioural intention. Cues to action have been described as strategies to activate “readiness” where how-to information is provided to promote awareness and action [41]. As such, it points more to external factors available to the individual, which is no longer within our study’s scope. In comparison to the general population, due to the door to door random sampling method, the study respondents were younger and slightly skewed towards females. However, neither age, gender, nor ethnicity influenced the predictors of vaccine uptake. Participants with higher education level were significantly more likely to take the influenza vaccine but no significant relationship was found between educational level and intention to take the dengue vaccine. Response rates were low, which could affect respondent views. However, a broad range of respondents were sampled from all over the country to lessen this bias. Intention to vaccinate does not always lead to actual behaviour; however, it is the best estimate of future vaccination. There could be many other factors influencing individuals’ vaccine uptake intentions. Future research should utilize other theoretical frameworks such as Theory of Planned Behaviour and Protection Motivation Theory to examine if the predictive power of the model is stronger.

## 5. Conclusions

In conclusion, this study proposes practical implications for improving vaccination uptake in Singapore. Despite perceived differences in the predictors of vaccine uptake between the diseases, intention to uptake rates are similarly low. This suggests that more work needs to be done to improve vaccination uptake at an intrinsic level, with a focus on highlighting the predictors of vaccine uptake discussed in this paper. More specifically, programs conducted for public health promotion should focus on increasing the public’s awareness of the susceptibility towards the diseases, seriousness of the diseases, and benefits of getting vaccinated. Public health communication campaigns should take these factors into consideration when developing policies and strategies for dengue and influenza prevention. The results of the study can also be used to monitor the efficiency and improvement of health promotion programs and behaviours of the public. Hence, the research also acts as a baseline study for research on two major diseases, influenza and dengue.

## Figures and Tables

**Table 1:** Demographics.

Demographics	*n* (%)
*n* = 1000	
Age (years)	
21–30	250 (25)
31–40	196 (19.6)
41–50	195 (19.5)
51–60	212 (21.2)
61–70	147 (14.7)
Gender	
Male	387 (38.7)
Female	613 (61.3)
Ethnicity	
Chinese	785 (78.5)
Malay	126 (12.6)
Indian	55 (5.5)
Others/No response	34 (3.4)
Highest Educational Attainment	
No formal schooling/PSLE or equivalent	189 (18.9)
GCE O/N/A level/Diploma	566 (56.6)
Degree/higher education	245 (24.5)

PSLE: Primary School Leaving Exam, GCE O/N/A: General Certificate of Education Ordinary/Normal/Advanced.

**Table 2:** Mean Comparisons of the predictors Between Influenza and Dengue.

	Influenza		Dengue		*t*	d*f*	*p*
	Mean	SD	Mean	SD			
Susceptibility	2.32	0.90	2.22	0.89	4.072	999	***
Severity	3.18	0.98	3.99	0.87	−25.306	999	***
Benefits	3.54	0.94	3.69	0.91	−5.909	999	***
Barriers	3.07	0.88	3.00	0.86	2.487	999	
Knowledge	2.87	0.87	3.17	0.84	−10.971	999	***

*** *p* < 0.001.

**Table 3:** Regression of Perceived Susceptibility, Severity, Benefits, Barriers, and Knowledge on the Likelihood of Getting Influenza Vaccine.

Influenza	*B*	S.E.	*β*	*t*
Susceptibility	0.293	0.037	0.246 ***	7.926
Severity	−0.086	0.039	−0.079	−2.230
Benefits	0.352	0.039	0.308 ***	8.947
Barriers	−0.037	0.038	−0.031	-0.988
Knowledge	0.082	0.037	0.067	2.245
R^2^			0.167	

*** *p* < 0.001. (*B* = unstandardised beta, S.E. = standard error, *β* = standardised beta, *t* = *t*-test statistic).

**Table 4:** Regression of Perceived Susceptibility, Severity, Benefits, Barriers, and Knowledge on the Likelihood of Getting Dengue Vaccine.

Dengue	*B*	S.E.	*β*	*t*
Susceptibility	0.305	0.034	0.278 ***	9.015
Severity	0.019	0.040	0.017	0.473
Benefits	0.343	0.037	0.321 ***	9.288
Barriers	−0.148	0.036	−0.131 ***	−4.128
Knowledge	−0.008	0.037	−0.007	−0.207
R^2^			0.18.9	

*** *p* < 0.001.
